# Transcriptional and Functional Studies of a Cd(II)/Pb(II)-Responsive Transcriptional Regulator(CmtR) from *Acidithiobacillus ferrooxidans* ATCC 23270

**DOI:** 10.1007/s00284-012-0117-4

**Published:** 2012-05-04

**Authors:** Chunli Zheng, Yanjun Li, Li Nie, Lin Qian, Lu Cai, Jianshe Liu

**Affiliations:** 1 College of Environmental Science and Engineering, Donghua University, Shanghai, 201620 People’s Republic of China; 2 School of Mathematics, Physics and Biological Engineering, Inner Mongolia University of Science and Technology, Baotou, 014010 People’s Republic of China; 3 School of Environment and Energy, Inner Mongolia University of Science and Technology, Baotou, 014010 People’s Republic of China; 4 School of Mathematics, Physics and Biological Engineering Inner Mongolia Key Laboratory for Utilization of Bayan Obo Multi-Metallic Resources: Elected State Key Laboratory, Inner Mongolia University of Science and Technology, Baotou, China

## Abstract

The acidophilic *Acidithiobacillus* *ferrooxidans* can resist exceptionally high cadmium (Cd) concentrations. This property is important for its use in biomining processes, where Cd and other metal levels range usually between 15 and 100 mM. To learn about the mechanisms that allow *A*. *ferrooxidans* cells to survive in this environment, a bioinformatic search of its genome showed the presence of that a Cd(II)/Pb(II)-responsive transcriptional regulator (CmtR) was possibly related to Cd homeostasis. The expression of the CmtR was studied by real-time reverse transcriptase PCR using *A*. *ferrooxidans* cells adapted for growth in the presence of high concentrations of Cd. The putative *A*. *ferrooxidans* Cd resistance determinant was found to be upregulated when this bacterium was exposed to Cd in the range of 15–30 mM. The CmtR from *A*. *ferrooxidans* was cloned and expressed in *Escherichia* *coli*, the soluble protein was purified by one-step affinity chromatography to apparent homogeneity. UV–Vis spectroscopic measurements showed that the reconstruction CmtR was able to bind Cd(II) forming Cd(II)–CmtR complex in vitro. The sequence alignment and molecular modeling showed that the crucial residues for CmtR binding were likely to be Cys77, Cys112, and Cys121. The results reported here strongly suggest that the high resistance of the extremophilic *A*. *ferrooxidans* to Cd including the Cd(II)/Pb(II)-responsive transcriptional regulator.

## Introduction

Cd is extremely toxic for all living organisms even when present at a low concentration. Cd^2+^ can easily enter bacterial cells by the transport systems for essential divalent cations such as Mn^2+^ or Zn^2+^, so almost all prokaryotes and eukaryotes have developed mechanisms to prevent excessive accumulation of Cd^2+^ in the cells. Usually, the operons encode metal responsive transcriptional regulators along with resistance proteins such as efflux pumps or transport proteins, reductases, and metal-sequestering proteins. In prokaryotes, the SmtB/ArsR and MerR family of metal sensor proteins represent two general classes of metalloregulatory proteins [2, 9].

*Acidithibacillus ferrooxidans* is a chemolithotrophic bacterium which is often subjected to different kinds of environmental stress such as temperature changes, presence of some toxic heavy metals or pH changes [6]. The tolerance of *A*. *ferrooxidans* to Cd is well documented in previous studies, which high to 10–100 mM. The uptake to Cd of *A*. *ferrooxidans* has also been reported, and a greater uptake was observed in tolerant strains [3]. Dopson et al. [7] used the in silico approach to detect genes involved in acidophile Cd resistance, found a number of Cd resistance operons are present in acidophile. However, the molecular mechanisms how these microorganisms to resist extremely high concentrations of Cd in their environment are still unclear.

By inspection of the full genomic sequence of *A*. *ferrooxidans* ATCC 23270, we found a putative Cd(II)/Pb(II)-responsive transcriptional regulator, and named as CmtR. However, no experimental data about the gene from *A*. *ferrooxidans* has been reported until now. We used RT-PCR detected the CmtR expression at transcription levels when *A*. *ferrooxidans* was cultivated with different concentrations of CdSO_4_. Furthermore, the gene of CmtR from *A*. *ferrooxidans* ATCC 23270 was cloned and successfully expressed in *E*. *coli*, finally purified by one-step affinity chromatography.

## Materials

*Acidithiobacillus ferrooxidans* ATCC 23270 was obtained from the American Type Culture Collection. A HiTrap chelating metal affinity column was purchased from GE healthcare LTD. DH5α competent cells, *E*. *coli* strain BL21 (DE3) competent cells were from Invitrogen Life Technologies. The Plasmid Mini kit came from Ambiogen Life Science Technology LTD. A gel extraction kit was obtained from OMEGA. *Taq* DNA polymerase, T4 DNA ligase and restriction enzymes came from MBI Fermentas of Germany. A QuikChange mutagenesis kit was from Stratagene. All other reagents were of research grade or better and were obtained from commercial sources.

## Methods

### Total RNA Extraction and cDNA Synthesis

Total RNA was isolated using the TRIzol^®^ reagent (Invitrogen Corporation, Carlsbad, USA) and purified with the RNeasy^®^ mini kit (Qiagen GmbH, Hilden, Germany) according to the manufacturer’s instructions, on-column DNase digestion was performed with the RNase-free Dnase (Qiagen GmbH, Hilden, Germany) to remove genomic DNA. The integral nature of total RNA was checked by 1.5 % agarose gel electrophoresis and ethidium bromide staining. Total RNA was quantified at OD260 and OD280 with NanoDrop^®^ ND-1000 spectrophotometer and then served as the template to synthesize cDNA.

### Real-Time PCR Detection

Real-time PCR primers were designed by using Primer Premier 5.0 and then synthesized by Sagon Biotech (Sagon, Shanghai, China). The forward primer is (5′–3′): CCCACTGGACGATTGC, reverse primer is (5′–3′): GCCTGTTTGGCTTACCG. Reference gene 16s forward primer is (5′–3′): TGGGTTCTAATACAATCTGCTA; reverse primer is (5′–3′): CGCATTTCACCGCTACA. The real-time PCR was carried out with iCycler iQ Real-time PCR detection system (Bio-Rad Laboratories, Inc., Hercules, USA): 1 cycle of 95 °C for 30 s, and then 40 cycles of 95 °C for 15 s, 55 °C for 30 s, and 72 °C for 30 s. At the completion of each run, melting curves for the amplicons were measured by raising the temperature 0.5 °C from 55 to 95 °C while monitoring fluorescence.

### Cloning, Expression, and Purification of the CmtR Gene from *A*. *ferrooxidans* ATCC 23270

The genomic DNA from *A*. *ferrooxidans* was used as a template for PCR reaction. The gene was amplified by PCR using primers that were designed to add six continuous histidine codons to the 5′ end of the amplified fragment. The sequence of the forward primer was 5′-CGCGCGAATTCAGGAGGAATTTAAAATGAGAGGATCGCATCACCATCACCATCACCGAATCGGCGCACTTGGACAG-3′. The sequence of the reverse primer was 5′-CTGCAGGTCGACTTAGCCTGTTTGGCTTACCGATCG-3′. PCR amplification was performed using *Taq* DNA polymerase, and samples were subjected to 30 cycles of 45 s of denaturation at 95 °C, 45 s of annealing at 62 0°C, and 2 min of elongation at 72 °C in a Mastercycler Personal of Eppendorf Model made in Germany. The PCR product was ligated into a expression vector pLM1, resulting in the plasmid pLM1::cmtR, and the plasmid was transformed into *E*. *coli* strain BL21 (DE3) competent cells for protein expression. The *E*. *coli* cells containing pLM1::CmtR plasmids were grown in rich LB (Luria–Bertani) medium to an attendance at 600 nm of 0.6 before isopropyl β-d-thiogalactoside (IPTG; 0.5 mM) were added to induce protein expression at 18 °C. The *E*. *coli* cells were harvested by centrifugation and were washed twice with sterile water. The purity of all the purified proteins was greater than 95 % as judged by electrophoresis analysis on a 15 % (w/v) polyacrylamide gel containing SDS followed by straining with coomassie blue.

### Cd(II) Binding Experiments

Metal binding experiments were carried out anaerobically at ambient temperature (24 °C) using a BioTek Synergy HT spectrophotometer. For Cd(II) titrations, apo-CmtR (0.8 mL of 245 μM) was diluted with a buffer (5 mM MES, 0.20 M NaCl, pH 7.0) and loaded into an anaerobic cuvette fitted with an adjustable-volume syringe loaded with 250 μL of Cd(II) titrant before removal from the anaerobic glovebox. Optical spectra of apo-CmtR and of the protein after each addition of a known aliquot (5–20 μL) of Cd(II) titrant were collected from 200 to 700 nm.

## Results and Discussion

### The Transcription of the CmtR in *A. ferrooxidans*

**Fig. 1:**
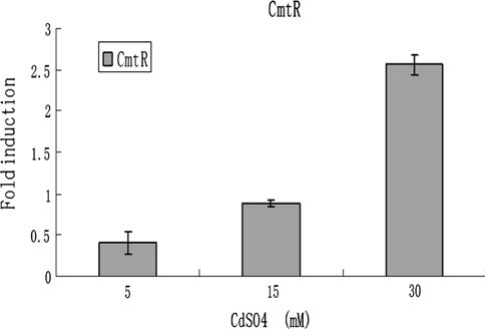
Expression of the gene CmtR in *A*. *ferrooxidans* after exposing to high concentrations of Cd. Fold induction of CmtR transcripts estimated by comparing transcript levels to those from cultures supplemented with no added Cd. The results were normalized against 16S rRNA to correct sample-to-sample variation. *Error bars* are s.d. from the mean of three independent samples

Previous studies have shown that the *A*. *ferrooxidans* had high Cd tolerance, it led us to hypothesis that if CmtR is involved in Cd transport and resistance, CmtR expression should be responsive to changes in Cd concentrations exogenously supplied to mycobacteria cell cultures. We incubated *A*. *ferrooxidans* cultures in 9K medium supplied with different concentrations of CdSO_4_, and we subjected these samples to RT-PCR (RT-PCR). RT-PCR experiments showed that gene encoded by CmtR is transcripted in *A*. *ferrooxidans* (Fig. 1). In the presence of 5 mM Cd^2+^, the CmtR expression by *A*. *ferrooxidans* was not significantly changed, suggesting a natural increase or decrease in the expression of these metal resistance genes under unfavorable growth conditions. In the presence of 15 mM Cd^2+^, the CmtR expression was upregulated about 1 fold. When the Cd concentration reached to 30 mM Cd^2+^, the CmtR expression showed a high level of induction about three fold, thus, tolerance concentration of 30 mM Cd^2+^ clearly had a significant effect on the metal resistance in this acidophilic bacterium. More importantly, transcript levels showed a strong Cd-dependent induction, which was consistent with the hypothesis that these genes participate in an organized response to Cd toxicity [8].

### CmtR from *A. ferroodoxins* Bound Cd Specifically

**Fig. 2:**
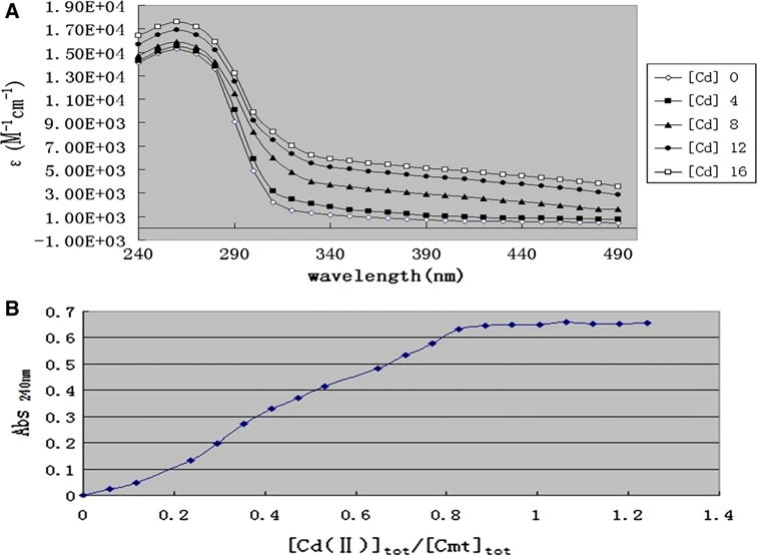
Binding of CmtR to Cd(II) monitored by optical absorption spectroscopy. **a** Absorption coefficient of Apo-CmtR-Cd(II) complex as a function of light wavelength at various ratio of [Cd(II)]/[CmtR] as indicated. **b** Absorption coefficient of Apo-CmtR–Cd(II) complex as a fraction of absorption coefficient of Cd–S bond plotted as a function of [Cd(II)]/[CmtR] at a fixed wavelength (240 nm)

The next challenge was to identify the inducer recognition sites of CmtR. Here, we were able to purify CmtR from *A. ferroodoxins* to homogeneity, the purity of the protein was examined by SDS-PAGE and single bands corresponding to the 17.4 kDa proteins were observed with >95 % purity (not shown), which was in agreement with the deduced molecular weight of CmtR. UV–Visible absorption spectra was used to detect any involvement of thiols in CmtR Cd(II) binding [5]. Results (Fig. 2a) showed a single strong absorption envelope with a maximum absorption of 19,000 M^−1^ cm^−1^ at ≈240–280 nm, indicating formation of the Cd(II)–CmtR complex. This intense absorption is assignable to S^−^ → Cd(II) ligand-to-metal charge transfer (LMCT) transitions, giving an absorption of є258 ≈ 5,500–6,000 M^−1^ cm^−1^ per Cd−S bond. These data are, therefore, consistent with at least three cysteinyl ligands involved in Cd(II) coordination. Meanwhile, any “non-thiolate” Cd(II)-binding residues that lack appreciable LMCT absorption would not be detected by this experiment. Binding of Cd(II) by wild-type CmtR, as monitored by optical spectroscopy, occurs with a stoichiometry of ~0.7 Cd(II) per CmtR monomer as shown in Fig. 2b.

### Identification of Cysteine Residues Essential for Cd(II)-Recognition

**Fig. 3:**
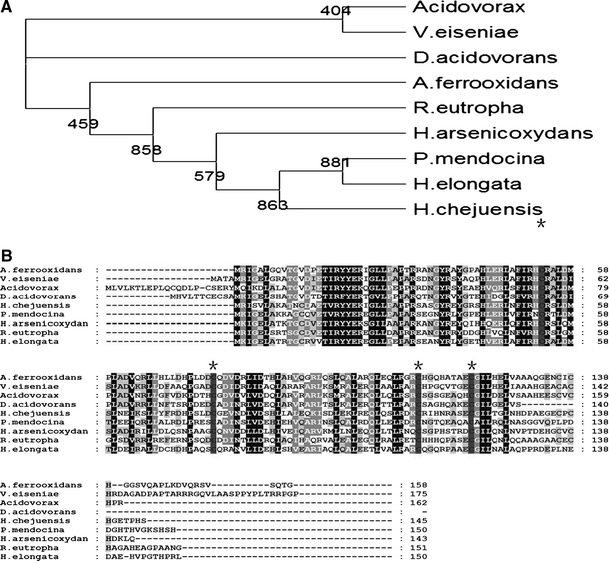
**a** An evolutionary tree of CmtR constructed from the amino acid sequences using ClustalX. The dendrogram (phylogenetic tree) describes the approximate groupings of protein sequences by similarity where the distance (% divergence) is calculated between every pair of sequences. A larger distance corresponds to more divergent sequences. **b** The sequence alignment of CmtR from *A*. *ferrooxidans* and other eight microorganisms. The strains for each organism are: *A*. *ferrooxidans*, ATCC 23270; *Verminephrobacter eiseniae*, EF01-2; *Acidovorax* *sp.*, JS42 D; *Delftia acidovorans*, SPH-1; *Hahella chejuensis*, KCTC 2396; *Pseudomonas* *mendocina*, ymp; *Herminiimonas arsenicoxydans*; *Ralstonia* *eutropha*, H16; *Halomonas elongate*, DSM 2581. The four residues proposed to coordinate metal binding are marked by *asterisk*

**Fig. 4:**
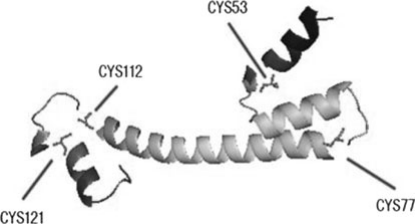
CmtR tertiary structure predicted by Swiss-Model and deduced coordinates of metal ion binding residuals of CmtR. CmtR is from *A.ferrooxidans*; the basis for model prediction is the crystal structure of the Zn(II)–ZntR complex of *E. coli*

Having established that Cd(II)-coordination in vitro involves thiols, the next question is which, if any cysteine residues are essential for inducer recognition and might supply these ligands? The evolutionary tree and sequence alignment of CmtR from *A. ferrooxidans* and other sources is shown in Fig. 3a, b. The CmtR from *A*. *ferrooxidans* contains six cysteine residues, the sequence alignment showed that four of them (Cys_53_, Cys_77_, Cys_112_, and Cys_121_) are strongly conserved. The tertiary structure of CmtR from *A*. *ferrooxidans* predicted by SwissModel using crystal structure of the Zn(II)–ZntR form of *E*. *coli* as the basis structure is shown in the Fig. 4 [1, 9]. ZntR from *E*. *coli*, belonging to the MerR transcriptional regulator family, is activated in the presence of Zn(II) and is partially responsive to Cd(II) and Pb(II). Mutagenesis experiments have shown that three cysteine residues in ZntR, known as Cys_79_, Cys_114_ and Cys_124_, bind directly to Zn(II) and that these residuals are essential to the correct function of the protein [4]. From the predicted tertiary structure, the Cd(II) binding sites of the CmtR are likely to be at Cys_77_, Cys_112_, and Cys_121_, similar to that of ZntR.

## Summary

We report here first the Cd(II)/Pb(II)-responsive transcriptional regulator from *A*. *ferrooxidans*. The result of transcriptional and functional studies showed that a Cd homeostasis mechanism including the CmtR is of importance for the survival of this extremophilic microorganism.
